# Current Developments of Analytical Methodologies for Aflatoxins’ Determination in Food during the Last Decade (2013–2022), with a Particular Focus on Nuts and Nut Products

**DOI:** 10.3390/foods12030527

**Published:** 2023-01-24

**Authors:** Andrea Schincaglia, Juan Aspromonte, Flavio A. Franchina, Tatiana Chenet, Luisa Pasti, Alberto Cavazzini, Giorgia Purcaro, Marco Beccaria

**Affiliations:** 1 Department of Chemical, Pharmaceutical and Agricultural Sciences, University of Ferrara, Via L. Borsari 46, 44121 Ferrara, Italy; 2 Laboratorio de Investigación y Desarrollo de Métodos Analíticos, LIDMA, Facultad de Ciencias Exactas, Universidad Nacional de La Plata, CIC-PBA, CONICET, Calle 47 Esq. 115, La Plata 1900, Argentina; 3 Department of Environmental and Prevention Sciences, University of Ferrara, via L. Borsari 46, 44121 Ferrara, Italy; 4 Gembloux Agro-Bio Tech, University of Liège, Passage des Déportés 2, 5030 Gembloux, Belgium; 5 Organic and Biological Analytical Chemistry Group, MolSys Research Unit, University of Liège, 4000 Liège, Belgium

**Keywords:** mycotoxins, aflatoxins, nuts, sampling, sample preparation, chromatography, direct techniques, aflatoxin determination

## Abstract

This review aims to provide a clear overview of the most important analytical development in aflatoxins analysis during the last decade (2013–2022) with a particular focus on nuts and nuts-related products. Aflatoxins (AFs), a group of mycotoxins produced mainly by certain strains of the genus *Aspergillus* fungi, are known to impose a serious threat to human health. Indeed, AFs are considered carcinogenic to humans, group 1, by the International Agency for Research on Cancer (IARC). Since these toxins can be found in different food commodities, food control organizations worldwide impose maximum levels of AFs for commodities affected by this threat. Thus, they represent a cumbersome issue in terms of quality control, analytical result reliability, and economical losses. It is, therefore, mandatory for food industries to perform analysis on potentially contaminated commodities before the trade. A full perspective of the whole analytical workflow, considering each crucial step during AFs investigation, namely sampling, sample preparation, separation, and detection, will be presented to the reader, focusing on the main challenges related to the topic. A discussion will be primarily held regarding sample preparation methodologies such as partitioning, solid phase extraction (SPE), and immunoaffinity (IA) related methods. This will be followed by an overview of the leading analytical techniques for the detection of aflatoxins, in particular liquid chromatography (LC) coupled to a fluorescence detector (FLD) and/or mass spectrometry (MS). Moreover, the focus on the analytical procedure will not be specific only to traditional methodologies, such as LC, but also to new direct approaches based on imaging and the ability to detect AFs, reducing the need for sample preparation and separative techniques.

## 1. Introduction

Aflatoxins (AFs) are toxic substances produced by certain species of fungi (moulds) found naturally worldwide and practically unavoidable. They can contaminate food crops and pose a serious health threat to humans and livestock. Indeed, their carcinogenic potential is well documented [1,2,3,4,5], and although some food processing practices (roasting, toasting, and heating) may reduce their presence in finished products, AFs are not degraded under normal cooking conditions [6]. It is noteworthy that climate change is predicted to significantly impact the spread of this threat [7,8]. In fact, although AFs contamination prevails both in warm humid climates and in irrigated hot deserts, different regions are expected to face increases in temperatures and experience drought, conditions that will promote AFs contamination even in temperate regions [8,9]. Hence, in light of climate change and the worldwide trade of food commodities, the control and monitoring of the presence of AFs and the AFs producers’ fungi are key to protect consumers’ health.

Moreover, besides the toxicological concern, these contaminants pose a significant economic burden, causing an estimated 25% or more of the world’s food crops to be destroyed annually [10]. For instance, in recent years, the exports of many nuts to the European Union (EU) market have faced problems due to the low limits for AFs set by the EU compared to other countries. For example, the EU limits are set at 8 µg/kg for AFB_1_ and 10 µg/kg for the sum of AFB_1_, AFB_2_, AFG_1_, and AFG_2_ for almonds and pistachios for direct human consumption [11], while in the USA, these limits are set at 15 µg/kg for the total AFs content in pistachios and peanuts [12,13]. In the last decade, there have been several risk notifications assessed by the EU Rapid Alert System for Food and Feed (RASFF) on US food and feed products contaminated with AFs and exported to the EU market. About 99% of these notifications were due to contamination in almond, peanut, and pistachio nuts. Moreover, a border rejection of the exported U.S. nuts was reported for more than 78% of AF notifications [14].

Since the discovery of AFs and the awareness of the threat these natural contaminants could bring, the scientific community has been working hard to develop sensitive, robust, and reliable analytical procedures, following the scientific trends, the updated risk assessment, the regulation requirements, and the technical instrumental development [15]. In fact, institutions progressively reduced the maximum admitted levels for AFs in foodstuffs accordingly to new toxicological findings. Additionally, these findings are possible thanks to the increased sensitivity of the newer analytical methods [16].

This review aims to provide a general overview of the issue of the AFs problem, with an emphasis on nuts because of their high economic value and because they are among the most affected commodities. Although numerous contributions have been published on AFs in food and feed, to the best of our knowledge, a detailed overview of the occurrence and the main analytical developments in AFs investigation has not yet been reported. Accordingly, the most common methods for the analysis of AFs in nuts will be discussed, focusing on developments of the last decade, aiming to provide a resourceful analytical guide for this challenging field.

The discussion will start framing the problem in its toxicological and legislative aspects. Next, the cumbersome issue of sampling will be addressed before entering the core of the review regarding the analytical determination. In fact, up to date, sampling is still the largest source of variability associated with the mycotoxin tests [17]. Although there exist sampling plans compiled by competent authorities, as in the case of the EU [18], the inhomogeneous distribution of contaminated kernels in a batch can significantly affect the final analytical result [17,19]. A discussion will be further held to investigate the different procedures applied to the sample preparation, starting from the homogenization, in which different grinding techniques can be used (dry or cryogenic grinding and slurry mixing), to purification and extraction involving more traditional approaches such as partitioning and innovative techniques using microwave or immunoaffinity. The aim of the sample preparation step is indeed to obtain an extract that is suitable for analysis and enriched with the target analytes. As sample preparation represents an important step of the analytical workflow, and considering the general trend towards more selective, rapid, and potentially eco-friendly procedures, a dedicated section discussing this crucial step is reported.

In the end, a comprehensive overview of the analytical methods employed is given, divided into indirect (separative methods, i.e., chromatographic) and direct methods (e.g., ELISA and imaging). Indirect techniques can be briefly summarized as chromatographic methodologies coupled to suitable detectors (namely mass spectrometry and/or fluorescence detector), in which expertise are required to obtain the desired data. Direct methodologies, with the exception of ELISA, are rather new and, despite being still immature, their applicability has been investigated to provide a rapid analysis from which food companies could benefit.

## 2. Aflatoxin’s General Information

### 2.1. Formation and Occurrence

**Figure 1:**
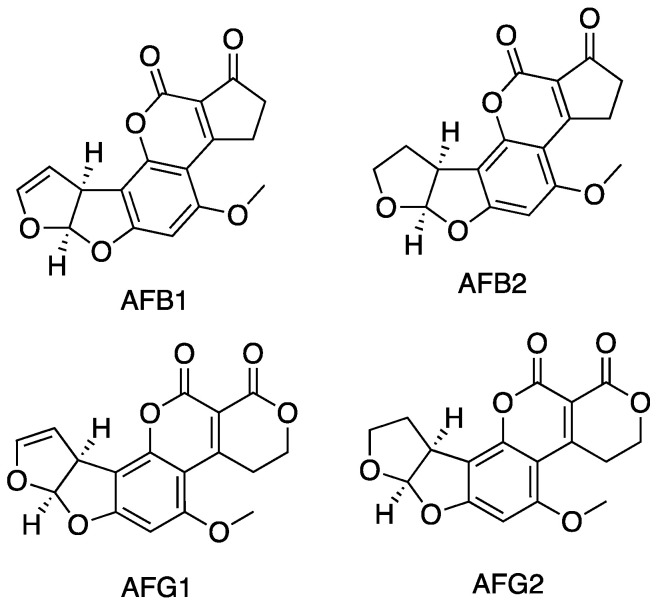
Chemical structure of the main aflatoxins: AFB_1_, AFB_2_, AFG_1_, AFG_2_.

Mycotoxins are low molecular weight compounds (usually less than 1000 Daltons), naturally occurring and practically unavoidable [5]. Specifically, the term mycotoxin refers to hazardous chemicals synthesised by fungi infesting crops [20,21]. Among them, AFs have been identified for the first time in Great Britain in the 1960s, and since then great efforts were made to study and analyse this class of compounds [22]. AFs are difuranocoumarin derivatives, which consist of five heterocycles. A bifuran group is attached to one side of the coumarin nucleus, while another cyclic group is attached to the other side of the coumarin nucleus. Two main groups of AFs are defined according to the nature of this ring, namely AF B-type (pentanone ring) and AF G-type (six-membered lactone ring). Their chemical structure is presented in Figure 1.

These toxins are secondary metabolites produced mainly by fungi from the genus *Aspergillus flavus* and *A. parasiticus*, although they are also produced by other species of *Aspergillus* as well as by *Emericella* species. *A. parasiticus* can produce both B-type (AFB_1_, AFB_2_) and G-type (AFG_1_, AFG_2_) AFs [23], while *A. flavus* has been thought to produce only B-type AFs. However, a recent study reported that some Korean strains of *A. flavus* can also produce G-type AFs [24].

The AFs biosynthesis occurs through complex pathways, involving many genes and enzymes [25]. *Aspergillus* species that produce AFs can colonize many crops in the field, during harvest, in storage, and during processing. Nevertheless, the highest level of contamination generally occurs during the post-harvest spoilage of food products stored under non-appropriate conditions, such as high-water activity or at a beneficial temperature for AFs to be synthesised [26]. Indeed, this kind of contamination generally takes place in tropical and sub-tropical climate areas, although it has been predicted that climate change could favour the spread of these AFs-producing fungi also in temperate climate areas [23]. So far, more than 20 AFs are known, and most of them have been described as mammalian biotransformation products of the main ones [27].

### 2.2. Toxicity and Legislation

Various foodstuffs can contain AFs, such as nuts, cereals, spices, oilseed crops, milk and dairy products, and other foods of animal origin [28]. AFs can be produced on cereals (with rice and corn being the most contaminated) in the field as well as during storage, involving the whole plant and/or the grain. Although spices represent a favourable matrix for the growing of mould-producing AFs, the level of contamination is generally lower than in cereals. In milk and dairy products, AFs contamination is proportional to the number of contaminated feeds that mammals ingest. After ingestion, most of the AFs (mainly AFB_1_ and AFB_2_) are eliminated through urine and faeces, and only a small fraction is bio-transformed in the liver. However, this bio-transformed fraction is excreted together with milk, in the form of AFM_1_ and AFM_2_ (from AFB_1_ and AFB_2_, respectively). It has been estimated that the ratio of ingested AFB_1_ to excreted AFM_1_ (in milk) is around 1–3% [29]. Residues of AFs and their metabolites can also be present in other foods of animal origin, such as meat and eggs [28,30].

Oilseeds crops mainly used to produce cooking oils, protein meals for livestock, and industrial uses, such as soybeans, sunflower seed, canola, rapeseed, safflower, flaxseed, mustard seed, peanuts, castor beans, sesame, pistachio, and cottonseed, are susceptible to AF contamination. Among them, peanuts are the most susceptible [28]. Fungi proliferation on peanuts plants and the subsequential nuts contamination represent a serious food safety concern in peanut-producing regions worldwide [31], causing a significant economic issue due to the high unit value of this crop, besides the health problems. In fact, import into the European market is impacted by the stricter AFs’ regulations (lower admitted limits) than those in some of the producing countries [11,18,32].

Only the presence of a few AFs is constantly monitored in food commodities due to their carcinogenic potential; among these, there are AFB_1_, AFB_2_, AFG_1_, AFG_2_, and two metabolites (AFM_1_ and AFM_2_, formed from AFB_1_ and AFB_2_ respectively). AFB_1_ is the most frequently found AF in food samples. It is also considered the most potent carcinogen found in nature. Thus, it is classified, together with AFG_1_, as group 1 (carcinogenic to humans) by the International Agency for Research on Cancer (IARC). Although there is limited evidence for the carcinogenicity of AFB_2_ and inadequate evidence for the carcinogenicity of AFG_2_, these two AFs are also classified as group 1 by the IARC [6,33]. The carcinogenic potential of AFB_1_ and AFG_1_ is due to the formation of toxic metabolites after food ingestion. After first-pass metabolism in the liver, the double bond in the furan ring of both AFB_1_ and AFG_1_ can be oxidized to form a reactive epoxide that can easily produce adducts with DNA [34]. AFM_1_ is a principal hydroxylated-AFB_1_ metabolite (as well as AFM_2_ for AFB_2_). It is bio-transformed by hepatic microsomal cytochrome P450 in cows fed with products contaminated with AFB_1_ [35]. In experimental animals, AFM_1_ demonstrated a carcinogenic potential; thus, it is classified as carcinogenic to humans (group 1) by the IARC [33,34].

The AFs exposition can occur by consuming contaminated plant products (such as pistachios, peanuts, corn, rice, etc.) and meat or dairy products from animals that have eaten contaminated feed. There are three in vivo metabolites of AFB-1 that have been validated as biomarkers of exposure to dietary intake of AFs: AF-alb (AFB_1_-lys), urinary AF-N7-gua, and urinary AFM_1_ AF-alb (AFB_1_-lys). In general, AF-alb reflects longer-term exposure (i.e., several weeks), while urinary AFM_1_ and AF-N7-gua are normally used as biomarkers to determine recent exposure [34]. The human AFs dietary intake can be dissimilar between developed and developing countries, with about 1 ng/kg body weight per day in the first case and about 100 ng/kg body weight per day for the latter [34,36].

In the last decades, the Joint Food and Agriculture Organisation of the United Nations (FAO) and World Health Organisation (WHO) Expert Committee on Food Additives (JECFA) performed various risk assessments and evaluated many epidemiological studies, estimating the cancer potencies of AFs. Although this estimation is subjected to many variables such as diet, health status, gender, duration of exposure, and individual characteristics in general, the maximum level for AFs was set following the principle of “as low as reasonably achievable”, considering the outcome of the risk assessment and the analytical capabilities [34]. Following the European Food Safety Authority (EFSA) scientific opinions, both sampling procedures and maximum limits have been updated over time accordingly in Europe [34,37,38]. The latest EFSA opinion revising the human health risks related to the AFs contamination in food based on new exposure data was published in 2020 [34], confirming the previously published outcomes (2007), where the genotoxicity and carcinogenicity of these substances were established [39].

In Europe, limits are provided for the total AFs in food (sum of AFB_1_, AFB_2_, AFG_1_, and AFG_2_) and for the concentration of AFB_1_ itself due to its high carcinogenicity. Nowadays, the maximum levels of AFs in food and the sampling procedures in Europe are set by the European Commission Regulations (EC) No.1881/2006 and 401/2006, and subsequent amendments [11,18]. In general, due to the different requirements (feasibility and economic impact) and approaches to risk analysis, there is still a large variability among the different regulatory bodies around the globe [10].

In addition, AFs contamination represents an even more severe issue in developing countries [36]. Indeed, the difference in countries’ risk perception, data collection, control approaches, and risk assessment models create disparities between countries in terms of AFs regulatory limits. Developed countries with better scientific and technical know-how often tend to adopt lower regulatory limits than those set by the global food safety regulatory body, namely the WHO/FAO joint Codex Alimentarius Commission [32].

## 3. Sampling

Although often overlooked, sampling is a crucial stage in every analytical procedure, but this is particularly cumbersome when dealing with mycotoxins. It has been estimated that in nut analysis the sampling accounts for up to 75% of the total variance associated with the whole analysis, due to the inhomogeneous distribution of contaminated kernels in a batch [17,19].

The total variability of the sampling method can be reduced by increasing the size of the sample or the subsample and increasing the number of aliquots quantified by the analytical method, thus reducing the number of batches misclassified by the sampling plan [18]. In 1993, a FAO technical consultation evaluated 35 sampling protocols providing important recommendations for sample collection and preparation procedures [40]. The FAO also developed in 2013 an online, free access mycotoxin sampling tool that can provide support in analysis performance of sampling plans and determining the most appropriate plan to meet users’ defined objectives. Many of the recommendations proposed by the FAO in 1993 have been integrated into the European Union Commission Regulation (EC) 401/2006 which sets the methods for sampling and analysis for the official control of the level of mycotoxins in foodstuff [18]. This regulation is extremely detailed and provides sampling methodologies for different foodstuffs according to the weight (or volume) of the considered lot.

## 4. Analytical Methodologies

Analytical methodologies regarding the analysis of AFs can be classified between direct and indirect methods. Both are widely used in food analysis, and the choice between them is strongly connected to economic factors, the purpose of the analysis, and the number of samples to be analysed.

**Figure 2:**
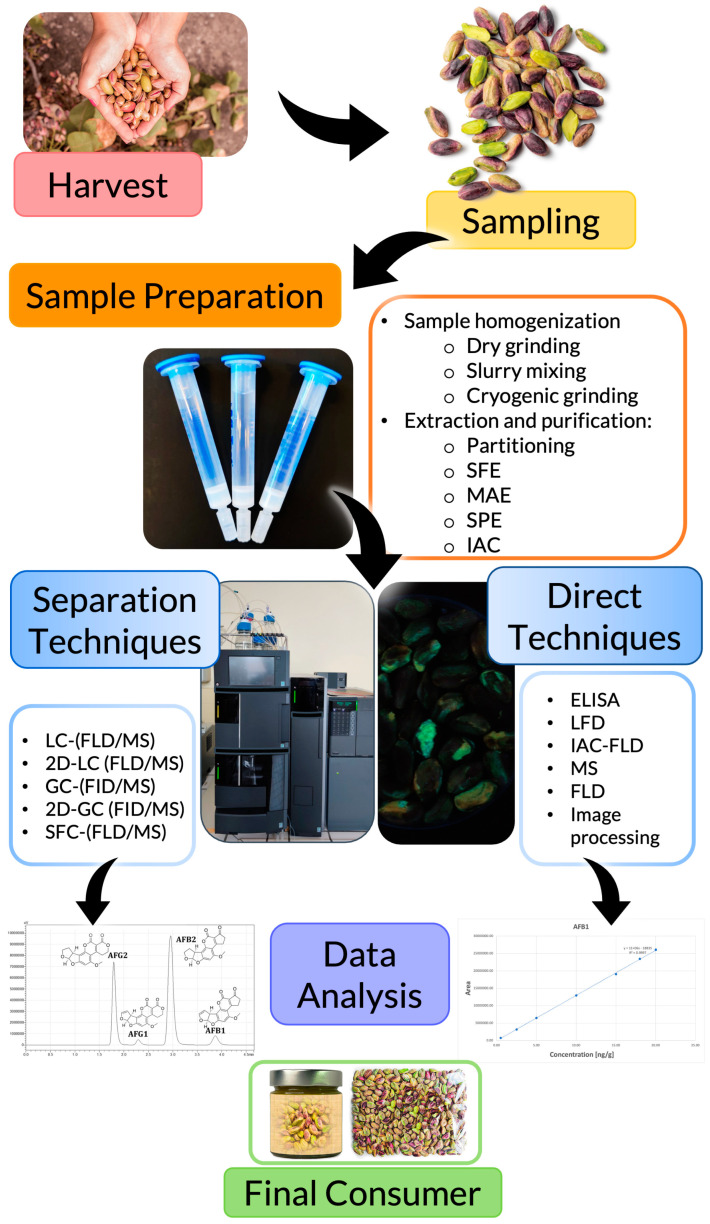
Workflow of AFs’ analysis in nuts: harvest, sampling, sample preparation, analytical methodology (direct, semi-direct, indirect), data analysis, and the final consumer.

Indirect analysis has to first undergo sample preparation, followed by separation of the analytes of interest (between them and from the matrix) and detection. On the other hand, direct approaches go from sample preparation directly to detection. It is worth mentioning that direct analysis could be subdivided into semi-direct and direct. The first one requires a sample preparation step, while the second one does not need any pre-treatment. Nevertheless, due to the high complexity of the sample matrix, most of the time, a sample preparation step is required to overcome matrix effects and obtain accurate results. Strictly direct techniques that do not need any sample preparation are very recent and require further research to overcome some limitations before their full implementation for routine testing. In the following paragraphs, sample preparation, as well as indirect and direct (divided into semi-direct and direct) methodologies, are discussed (Figure 2).

### 4.1. Sample Preparation

As aforementioned, most of the analysis variance can be caused by inhomogeneous samples; therefore, sample homogenization is a key step after the sampling to ensure a representative analysis. Moreover, due to the complexity of food matrices, an extraction and purification pre-treatment is usually required as part of the analytical procedure.

#### 4.1.1. Sample Homogenization

Inhomogeneous samples can present pockets of higher concentration of AFs than the whole lot, or AFs may be locally absent. Therefore, the homogenization phase after sampling is fundamental to avoid misestimations or false negative results. The easiest way to obtain a homogeneous sample is through blending. There exist different blending techniques that can be applied depending on the sample nature. In general, the AOAC International Procedure 977.16 suggests that the sample must be blended enough to pass through a No. 20 sieve despite the chosen technique [41].

##### Dry Grinding

Dry grinding is the easiest and cheapest blending procedure to obtain a homogeneous sample. This technique has been widely applied for sample homogenization for AFs analysis in different food commodities, such as rice, cereals, maize, coffee beans, etc. [42,43,44]. However, the heat generated by the friction of moving blades can be an issue for nut samples. The fat and sugar content of these samples leads to the formation of clusters during the grinding process, resulting in particles bigger than the starting material. Moreover, besides the possibility to reduce homogeneity due to the formation of these clusters, cleaning the mill after every use can be laborious [44]. Furthermore, due to the fine-flowing particulate matter obtained, this technique presents a higher risk of respiratory exposure to AFs by the analyst than other alternatives [45].

##### Wet Grinding–Slurry Mixing

An interesting alternative to overcome the aforementioned limitations and risks of dry grinding is the use of slurry mixing or wet grinding procedures [45]. This kind of method has been a common practice for the extraction of AFs for many years. Since 1983, the Agricultural Marketing Service (AMS) of the U.S. Department of Agriculture suggests using this kind of methodology, and it has been later approved by the AOAC [46].

In the wet grinding process, the material is dispersed in a liquid phase, creating a homogeneous slurry product that cannot be achieved by dry grinding. Spanjer et al. evaluated homogeneity in terms of coefficients of variation comparing dry and wet grinding procedures [47]. They concluded that the lowest possible coefficients of variation are obtained for slurry mixing with 10 to 30 kg of sample. In this way, sampling errors and false-positive or false-negative results can be reduced to a minimum. Nevertheless, since it is not feasible for every laboratory to work with such large quantities of samples, analytical variances were also evaluated for smaller samples, namely 25 to 50 g. Kumphanda et al. showed that slurring 250 g and testing 25 g slurry mass has a greater effect on improving precision than increasing test portion mass from 12.5 g dry grind to 50 g dry grind [48].

Wet grinding can greatly influence the subsequent extraction process. Hence, identifying the optimal solvent to sample ratio or the kind of solvent to be used for the wet grinding process can enhance the extraction efficiency. Due to the polarity of AFs, polar solvents such as methanol, water, acetonitrile, and chloroform have been widely used for their extraction. An interesting work evaluating these parameters has been conducted by Cole and Dorner, who evaluated the extraction of AFs from naturally contaminated peanuts with different solvents and solvent to peanuts ratios [49]. They found no significant differences between the evaluated solvents and the solvent:sample ratios for samples contaminated with low concentrations of AFs (10 µg/kg). However, for samples contaminated with higher concentrations (100 or 1000 µg/kg), they concluded that the best options were 80:20 methanol:water (with 3:1 mL solvent:g sample) and 90:10 acetonitrile:water (with 2:1 mL solvent:g sample). The 80:20 methanol:water solvent composition would be the preferred alternative due to economic and sustainable considerations. A few years earlier, Whitaker et al. performed a series of experiments evaluating different ratios of methanol:water as solvent and solvent:peanuts proportions, concluding that a combination of 60:40 methanol:water solvent and 10.8 mL solvent:1 g peanut has the best extraction efficiency [46]. In 1998, the AOAC approved a method for the detection of AFs in shelled peanuts (AOAC Official Method 998.03) that uses 283 mL of 77% methanol in water solution to extract AFs from 196 g of sample [50].

Therefore, wet grinding can improve the recovery and precision of AFs quantitation compared to dry grinding, while reducing the respiratory exposure to AFs by the analyst [45].

##### Cryogenic Grinding

Cryogenic grinding can be extremely effective to treat dry fruits containing high oil content, such as nuts. As previously mentioned, heat produced by the grinding process can lead to clogging and buttering of the sample with the result of having a non-homogeneous sample and particles bigger than the starting material. Cryogenic grinding is carried out at low temperatures with a frozen sample. Thus, this technique requires a shorter grinding time and protects hot-labile components [51]. Most important, it provides extreme fine grinding and a high level of homogeneity, with smaller and more uniform particle sizes. Nevertheless, it requires specific grinding equipment and liquid nitrogen or dry ice as cooling agents, hence increasing the costs. Moreover, samples need to be kept in a refrigerator overnight before grinding, and after grinding, they need to be equilibrated to room temperature before further manipulation.

The possibility to obtain an extremely homogeneous distribution of fine and uniform particles is of particular interest for the analysis of AFs [52].

#### 4.1.2. Extraction and Purification

Depending on the food matrix treated, the extraction and the clean-up steps can be merged into a single step. However, when analysing solid samples, the two steps are usually separated. It is commonly known that the identification and quantification of target compounds in food can be challenging, due to the complexity of the matrix itself. Focusing on nuts, the extraction and purification steps are mandatory for having a sample free from interferences from the matrix. The matrix effect, i.e., the effect on an analytical assay caused by other components of the sample except for the specific analyte, could lead either to a loss in response, resulting in an underestimation of the amount of analyte, or an increase in response, producing an overestimated result. Focusing on the safety of food products, affected by AFs’ contamination, the choice of a proper extraction/purification procedure is indeed crucial to provide a reliable analytical result. Several extractions and purification techniques have been developed over the years, leading to rather reliable results. The choice of one over the other is mainly due to the availability of the instrumentation and the costs of analysis.

In the following section, an overview of the extraction and purification techniques used for nuts analysis is reviewed.

##### Partitioning

Since the discovery of these classes of mycotoxins in the late 1960s, partitioning has been widely applied for sample preparation for AFs analysis. Using a polar (or medium polar) solvent to extract AFs and then washing several times, the polar phase with a non-polar solvent is a useful and cheap methodology to concentrate AFs (in the polar solvent) and eliminate co-extracts [53]. Nevertheless, this approach is not able to purify and concentrate AFs adequately because many matrix interferences cannot be eliminated, and a subsequent clean-up step is mandatory to perform the analysis. As polar molecules, AFs are soluble in polar organic solvents such as methanol, chloroform acetonitrile, etc. The optimization of the extraction conditions needs to consider not only various combinations and ratios, of organic solvents and samples, but important consideration of the AFs stability in the extracted mixture should be made [54].

Among all organic solvents, chloroform is the most chosen one for AFs’ storage, despite its volatile characteristics. In fact, chloroform is a solvent with intermediate polarity in which AFs can reach good solubility. Moreover, AFs’ solution in chloroform shows a high degree of stability when stored at 4 °C. Nevertheless, in a recent study, Kiseleva et al. confirmed the stability of acetonitrile or methanol standard solutions of AFs stored at −18 °C for at least 14 months [55]. However, when it comes to water solutions, the stability of AFs is not granted and depends on the water:organic solvent ratio, AFs’ chemical structure, storage temperature, and the surface of the glass vials used to prepare the solution. Diaz et al. evaluated the degradation kinetics of AFs during 24 h; they found that the presence of at least 20% organic solvent and keeping the solution at refrigerated temperature (5 °C) in a treated vial ensured the stability of the AFs. The authors proposed the use of glass autosampler vials that were silanized or etched with 50% nitric acid. The latter probably helped to remove the glass surface contaminants that induce AFs transformation and/or adsorption [56].

In the AOAC Official Method of Analysis, several partitioning procedures are reported for AFs in peanuts, peanut butter, pistachio, pistachio paste, corn, and related products. These procedures are employed before the clean-up step. For example, the AOAC Official Method 991.31 for the detection of AFs in corn, raw peanuts, and peanut butter prescribes the use of a mixture of methanol, water, and sodium chloride to produce a slurry that is then further cleaned prior to analysis [57]. Briefly, a 25 g portion of sample is placed into a blender with 5 g of sodium chloride and 125 mL of an extraction solvent composed of 70:30 methanol:water. After blending for 2 min at high speed, the slurry mix obtained is filtered through a pre-folded filter paper. A total of 15 mL of this extract is then diluted with 30 mL of water and filtered again with a glass microfiber paper. The next clean-up step involves the use of an immunoaffinity column (IAC). Interestingly, Choochuay et al. determined AFB_1_ in feedstuff (including peanuts) using a quick easy cheap effective rugged safe (QuEChERS) method without the need for an additional clean-up step, followed by high-performance liquid chromatography (HPLC), with pre-column derivatisation and a fluorescence detector [58]. This approach showed good recoveries for peanuts, ranging between 93.89% and 109.37% with intra-day %RSDs < 5.89% and inter-day %RSDs < 2.47%. Moreover, they obtained a LOD of 0.6 ng/g and a limit of quantification (LOQ) of 0.9 ng/g for AFB_1_.

Considering the large amount of solvents required for traditional liquid–liquid extractions (LLE), this technique cannot be considered as environmentally friendly. Therefore, to minimize solvent consumption, while increasing concentration factors, liquid–liquid microextraction (LLME) and dispersive LLME (DLLME) have been widely applied for the determination of AFs in the last decade. DLLME is a two-stage process that allows the extraction of analytes and solutes in an aqueous phase, with the use of a modest quantity of organic solvents (extracting and dispersing solvents). The two steps are as follows. (1) The mixture of extracting and dispersing solvents is rapidly injected into a water sample. Thanks to the large surface area between the extracting solvent and the aqueous sample, the equilibrium state is quickly achieved. (2) Centrifugation is performed to break the dispersion and the coalesced organic phase is removed with a micro-syringe [59]. Most of the publications related to these approaches for mycotoxins focus on products such as milk and oil [51]. However, when dealing with a solid phase, grinding and a preliminary extraction step are required before the actual DLLME [60]. Hence, only a few DLLME procedures have been implemented for nuts and DLLME has been used as a clean-up step after partitioning. For instance, Arroyo-Manzanares et al. proposed a DLLME procedure for nuts samples, where the acetonitrile phase obtained after partitioning was evaporated under a gentle stream of nitrogen and reconstituted with 1 mL of methanol:water (50:50). Subsequently, 4 mL of water and 0.21 g of NaCl were added. The mixture of the disperser solvent (950 mL of acetonitrile) and the extraction solvent (620 μL of chloroform) was rapidly injected into the test tube with a 2 mL syringe. At this step, the AFs were extracted into the fine droplets of chloroform. After centrifugation, fine particles were sedimented and the sedimented phase was removed using a 1 mL syringe. The recovered phase was evaporated to near dryness under a gentle stream of nitrogen and reconstituted with 1 mL of methanol:water (50:50). The solution was filtered and injected into an ultra (U)HPLC-MS/MS system. Recoveries for AFs in different matrices ranged between 65.8% and 99.3% with %RSD < 11% in all cases [61].

##### Supercritical Fluid Extraction (SFE)

Supercritical fluid extraction (SFE) rose during the last 40 years thanks to its various applications in multiple fields, including the food industry. SFE has many benefits, and it is considered a green technique. Indeed, the supercritical fluid can be reused for further extraction; the process uses non-toxic solvents such as CO_2_ and can be easily coupled with other technologies [62,63]. So far, CO_2_ is the most used solvent in SFE. Although it is the main greenhouse gas that contributes to global warming, its low critical conditions (30.9 °C and 7.38 MPa) make it perfect for this kind of extraction: it is non-toxic, affordable, and non-flammable [64]. CO_2_ is certainly an eco-friendlier choice compared to organic solvents. Nevertheless, it is non-polar and molecules with a relatively polar nature, such as AFs, are in general not suitable analytes for SFE. Therefore, proper adjustments in the pressure and/or temperature parameters, as well as the use of modifiers, are applied to obtain an increased solvation force towards AFs. Additionally, an increase in pressure leads to a higher fluid density and enhanced solubility of the solute [65]. Nevertheless, increasing pressure to a certain point may reduce the diffusivity of the SFE solvent and result in reduced contact with pores in the raw material, limiting the solute dissolution. However, higher temperatures can increase diffusivity and reduce solvent density, leading to an increased solvation power [66]. Finally, the use of polar modifiers (co-solvents) changes the polarity of the extractant, increasing the extraction yield of polar compounds [67]. However, when treating food matrices rich in fatty and non-polar interferents with such a technique, the level of co-extracts may be considerable. Finding a good SFE methodology to be applied to AFs’ extraction is not an easy task and the presence of co-extracts requires further clean-up stages. To date, the only contribution during the last decade using SFE to extract AFs in food (peanut butter) was reported by Ogura and Shad in 2016 [68].

##### Microwave-Assisted Extraction (MAE) and Pressurized Liquid Extraction (PLE)

Microwave-assisted extraction (MAE) and pressurized liquid extraction (PLE) require less solvent than traditional techniques, making them more efficient, faster, and greener alternatives [69]. Thanks to localized temperature and pressure, analytes of interest can migrate from the matrix to the solvent at a more rapid rate and with comparable, or even higher, yields than traditional extraction techniques [70]. Despite these significant advantages, their application for AFs analysis is limited. Only a handful of applications can be found regarding MAE and PLE in food products. However, in these few contributions, different food matrices have been investigated, such as grains and derivatives, peach seed, milk powder, corn flour, rice, and maize, but no nuts and nut products [71,72,73,74]. It should be noted that despite their high efficiency and throughput, these approaches are still erroneously perceived as high-priced equipment still limiting their application in routine testing, thus explaining the reduced number of publications found in this area.

##### Solid Phase Extraction

Solid phase extraction (SPE) is a widely used sample preparation technique for the isolation of analytes of interest from a mobile phase. First, the analytes are retained in the stationary phase, separating them from the matrix. Then, the analytes are collected by elution with an adequate solvent.

SPE was developed as a complement or replacement of LLE, becoming a widely used pre-treatment technique since its introduction in the 1980s [75]. Its use for the analysis of AFs has not been the exception, using mainly silica gel, C_18_, or hard powdered magnesia-silica gel (e.g., Florisil^®^) as stationary phases [76]. Some works in the literature are reporting SPE methodologies applied for sample clean-up [77,78], and some of these procedures have been applied specifically to nut matrices [79,80,81,82].

An evolution of the classic SPE approach is the matrix solid-phase dispersion (MSPD), also called the dispersive (D)SPE method. MSPD is an SPE-based extraction, which has been already applied to the pre-processing of a variety of samples [83]. The main advantage is to combine extraction and clean-up in a single step. Moreover, MSPD reduced the amount of solvent needed for sample extraction, simplifying the pre-treatment. Primary-secondary amine (PSA) and octadecylsilane chemically bonded silica (C_18_) are the most popular functional dispersants, with several types of homemade ones also used. However, the several MSPD functional dispersants are still relatively limited and expensive.

**Figure 3:**
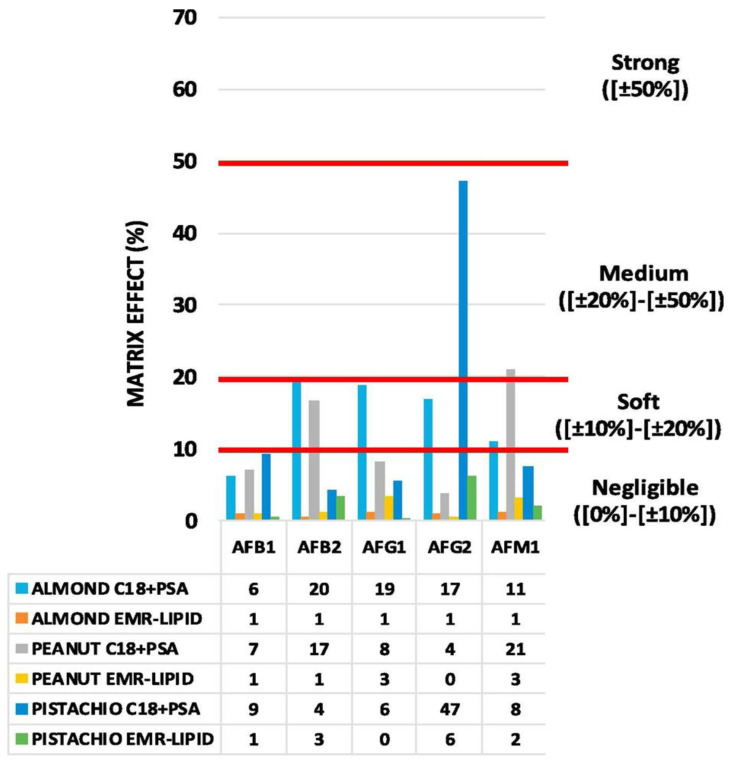
Matrix effects (%) obtained using different dispersive SPE sorbents in each sample studied. Modified and reprinted with the permission of [80].

In 2019, Wu et al. applied for the first time a deep eutectic solvent (DES)-MSPD methodology in the context of AF investigation in crops, including nuts. AFs quantification was carried out by HPLC coupled with a fluorescence detector (FLD). The intra-day and inter-day variability for AFs in all crop samples was less than 7.5%. Linearity was observed with R^2^ values (>0.994), with limits of detection (LODs) of 0.03–0.10 μg/kg and limits of quantification (LOQs) of 0.10–0.33 μg/kg [84]. In the same year, a similar approach for AFs determination was tested by Liang et al., coupling a DSPE (or MSDP) with UHPLC-MS/MS. After extraction of grinded chestnut samples, performed with a 10 mL solution of acetonitrile, water, and formic acid (79:20:1) for 5 min, the extract was blended with C_18_ powder as an adsorbent matrix. Recoveries ranged from 74.2% to 109.5%, with RSDs below 15% in all cases [85]. Alcántara-Durán et al. tested and compared an enhanced matrix removal-lipid (EMR-lipid) SPE sorbent material with a conventional mixture of primary secondary amine (PSA) and octadecyl-modified silica (C_18_) dispersive SPE sorbents for AFs extraction. Using an LC-MS/MS system, the authors showed that the EMR-lipid reduced the presence of matrix components more effectively, achieving a negligible matrix effect for all AFs studied in peanuts, pistachios, and almonds [80], as shown in Figure 3.

Recently, nanomaterials have received considerable attention due to their unique properties and application as promising absorbent structures in SPE. In 2022, an activated carbon-boron (AC-B) nanocomposite adsorbent material has been developed as a new SPE sorbent for AFs extraction in nuts. AFs detection and quantification were carried out with HPLC-FLD with post-column AF derivatization to increase sensitivity. The method was validated in terms of LOD, LOQ, linearity, reproducibility, and accuracy. The authors presented this methodology as an alternative to routine analysis due to several analytical and environmental advantages compared to the traditional immunoaffinity column methods [82]. In the same year, a novel modified magnetic SPE (MSPE) adsorbent material was synthesized by binding 3-aminopropyltrimethoxysilane and 1,8-bis (3-chloropropoxy) anthracene-9,10-dione to magnetic nanoparticles (MNP). MSPE was applied to the AFs extraction in hazelnut, peanut, and almond samples followed by HPLC-FLD analysis by post-column derivatization. The LOD for AFB_1_, AFB_2_, AFG_1_, and AFG_2_ was in a range of 0.15–0.05 μg/kg, with good linearity and precision, compared to routine methods [86]. Karami-Osboo et al. synthesized different adsorbent materials used in magnetic (M)DSPE for AFs B1, B2, G1, and G2 extraction at trace levels in nuts (pistachio) prior to determination by HPLC-FLD [87,88]. Taherimaslak et al. used a two-step extraction consisting of DLLME and vortex-assisted magnetic nanoparticle (VAMN)DSPE followed by surfactant-enhanced spectrofluorimetric detection for the determination of AFs in pistachio nuts, obtaining similar results compared to the AOAC standard method (IAC-HPLC-FLD) [89]. The use of an ultrasound-assisted matrix (UAM) SPD method was proposed for the first time by Manoochehri et al., combining the extraction method with HPLC-FLD for the determination of AFs in pistachios. The influence of different variables including the type of dispersing phase, the sample-to-dispersing phase ratio, the type and quantity of clean-up phase, the ultrasonication time and temperature, and the nature and volume of the elution solvent were investigated using a Box–Behnken design through response surface methodology and experimental design. Briefly, pistachios were blended with C_18_ until the mixture became homogeneous. The mixture was then located in a glass syringe and compressed. Then, acetonitrile was loaded while both ends of the cartridge were closed. The column was immersed in an ultrasonic bath at 30 °C for 11 min and the acetonitrile was collected. C_18_ showed the best characteristics as dispersing phase, while acetonitrile was the best elution solvent. Recovery was satisfactory, ranging from 74% to 78% for all AFs, with RSDs in the 6.5–8.6% range [90].

In a recent study, this technique was further exploited in peanuts matrices using fabricated ZnO nanoflowers as adsorbents for a dispersive solid phase microextraction (DSPME) coupled HPLC-FLD after sonication, leading to an AFs recovery extraction ranging from 93.8% to 105.1% with RSDs < 4% [91].

Solid phase microextraction (SPME) constitutes an interesting alternative to SPE. SPME allows sampling extraction and sample concentration in a single step and also is easy to automate [92]. The basic principle involved in SPME is to expose a pre-coated surface to the sample matrix of interest. The coating extracts the compound of interest, and the extracted compounds can be transferred to a dedicated analytical instrument [93,94]. However, its use for AFs analysis is limited, mainly applied for determining volatile organic compounds (VOCs) profiles by GC-based techniques. Georgiadou et al. used head-space (HS)-SPME-GC-FID/MS along with dedicated statistical elaboration to distinguish healthy and AF-contaminated nuts. The contamination was confirmed using traditional HPLC-FLD analysis as a confirmatory method. Differences between contaminated and clean samples were spotted due to the identification of specific C_8_ markers belonging to the chemical families of alcohols, ketones, aldehydes, and sesquiterpenes in contaminated samples. However, the authors claimed that the presence of these compounds could be related to common non-aflatoxigenic fungi and not only specifically to *A. flavus* [95]. Beck et al. used complementary static SPME and dynamic needle-trap SPE followed by GC-MS and statistical analysis for the analysis of general fungal growth, and volatile emission profiles at varying relative humidity levels for the differentiation of stockpiled almonds [96].

Originally, SPME was mainly coupled with gas chromatography (GC) to analyse volatile compounds but its use in combination with LC for semi- and non-volatile compounds has grown over the last years [97]. Amde et al. developed an SPME based on ionic liquid (1-hexyl-3-methylimidazolium hexafluorophosphate) functionalized zinc oxide nanorods for the analysis of AFB_1_, AFB_2_, AFG_1_, and AFG_2_ in foods, including nuts, prior to HPLC analysis. Satisfactory results were reported in terms of linearity, LOD, and LOQ (0.07 and 0.73 μg/kg for AFB_1_, 0.01 and 0.12 μg/kg for AFB_2_, 0.04 and 0.44 μg/kg for AFG_1_, and 0.02 and 0.18 μg/kg for AFG_2_), with intra- and inter-day RSDs (in the ranges of 3.9–4.7% and 6.9–8.4%, respectively). The authors claimed that this approach is easy, fast, cost-effective, and environmentally friendly and can be used for the analysis of AFs in various food and agricultural products [98].

SPME can also be used without coupling it to any separation techniques, directly hyphenated with a mass spectrometry (MS) system. Tsai et al. developed a method based on SPME coupled with carbon fiber ionization (CFI)-MS to detect the presence of trace AFB_1_ in peanuts [99].

##### Immunoaffinity Column (IAC)

Since the first development of immunoassays more than 60 years ago, the advantage given by the employment of antibodies has had a significant impact in the testing and diagnostic fields [100]. Immunoaffinity columns (IACs) have become very popular in food safety analysis thanks to their specificity and simplicity to use for routine testing, in particular for the determination of AFs [101]. The principle behind IAC is simple and relies on the selective binding of antibodies with the specific antigen. The initial sample is loaded on the IAC and AFs are retained by the antibodies, while the rest of the matrix components pass through the column. Then, a wash step is required to remove any impurities that remained in the column. Finally, a solvent able to disrupt the bond between AFs and antibodies is required to elute the purified toxins. The rapid and high-effective clean-up provided by this technique made it one of the most applied clean-up approaches when it comes to AFs’ analysis. The final extract is compatible with different quantitative techniques such as fluorimetry, LC-FLD, and LC-MS without the necessity of further sample treatment [101].

**Figure 4:**
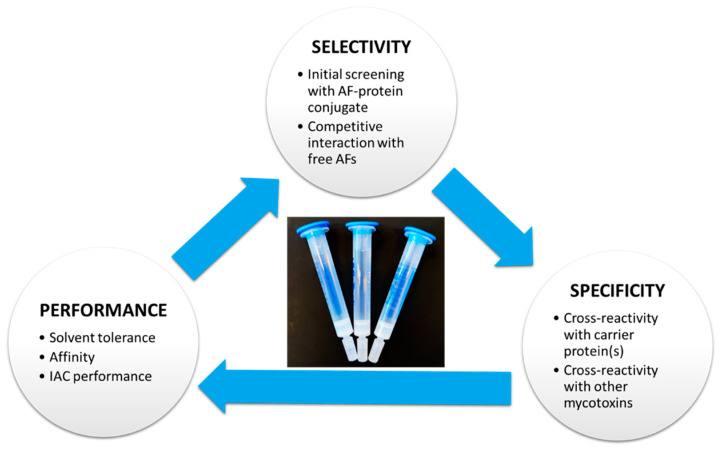
Scheme for AFs-specific antibodies to be used for immunoaffinity column development.

The specificity of the antibody should be evaluated so that it has an affinity towards AFB_1_, AFB_2_, AFG_1_, AFG_2_, and AFM_1_. However, besides antibody specificity, the selectivity and performance of the final method play an important role in the global evaluation and selection of the IAC method (Figure 4).

The satisfactory performances of clean-up methods based on IACs led to the publication of reference methods 991.31 and 999.07 by the AOAC International [57,102]. These procedures for the determination of total AFs in nut products establish the extraction of the test portion with methanol:water (either 80:20 or 70:30) in a blender. The extract is filtered on paper and diluted with water. The diluted solution is then loaded on an IAC containing monoclonal antibodies specific for aflatoxin B1, B2, G1, and G2. The syringe-barrel column is eluted with buffer solution, and AFs are isolated, purified, and concentrated on a column and removed from antibodies with methanol. According to the AOAC methods mentioned above, total AFs are quantified by fluorescence, using a 360 nm excitation wavelength and a 420 nm cut-off emission filter, and calibrated with total AFs standard solutions. Individual AFs are quantified by LC with fluorescence detection and post-column iodine derivatization. According to AOAC International Method 991.31 [57], an IAC for total AFs should be able to bind at least 80% of 5 ng AFB_1_, AFB_2_, and AFG_1_, and 60% of 5 ng AFG_2_. Dini et al. evaluated AFs contamination levels in pistachio nuts imported to the EU from Iran from November 2012 to October 2018 [103]. They used the AOAC Official Method 999.07 with minor modifications; an IAC was used to obtain purified AFs and the level of each AF was evaluated by RP-LC coupled to a fluorescence detector (FLD). The average recoveries of the spiked blank sample with 4 ng/g for AFB_1_ and AFG_1_ and 1 ng/g for AFB_2_ and AFG_2_ ranged between 78.6% and 97.6%, with RSDs below 8.5%. These values were within the acceptable parameters set by the European Commission [18].

An interesting aspect of using IACs is the possibility to have an automated system. This kind of system can be classified between off-line and on-line. When using an off-line system, the sample extract is loaded on the column, and washing and elution are carried out offline. The system subsequently makes an injection of the extract into the HPLC system. Once the parameters are set, the whole system can be run unattended. The first effort in this direction was conducted in 1991 by Sharman et al., who successfully modified a commercially available sample preparation system so that it can be used with an IAC [104]. Such a system was then employed for AFs analysis in a wide range of food commodities, including nuts. Further evolution led to a commercial robotic system for AFs analysis for an even wider range of food matrices. Carman et al. tested this system obtaining results comparable with other manual and official procedures for the determination of AFs in nuts. In the on-line approach, the IAC is directly connected to the HPLC or LC/MS through a system of switching valves [105]. Working in this direction, Rhemrev et al. were able to implement an on-line IAC system using reusable immunoaffinity columns. The system selects an immunoaffinity cartridge from a tray and automatically loads the extract. The cartridge is washed, and AFs B1, B2, G1, and G2 are eluted and transferred inline to the LC system for quantitative analysis using fluorescence detection with post-column derivatization. Each immunoaffinity cartridge can be used up to 15 times without loss in performance. The system showed recoveries higher than 80% for spiked samples of nuts. These results are comparable to manual procedures in terms of clean-up efficiency [106]. Recently, Dhanshetty et al. evaluated a similar method for AFs analysis, using an automated clean-up and HPLC analysis system with post-column derivatization (bromination). The method was evaluated against the conventional workflow using manual clean-up and HPLC analysis. The automated system provided better precision (RSD < 9%) than the conventional manual workflow (RSD 12–15%) [107].

Despite the great advantage of automated IAC clean-up to reduce laboratory manipulations while maintaining good results, its use is not widespread among laboratories yet for either off-line or on-line modes. Such procedures require specialized personnel and deep knowledge of the automated device. Although the clean-up step (and analysis) is completely automated, extraction still has to be conducted manually due to the high complexity of food samples. However, this implementation provides better quality control (QC) system, minimizes human errors, improves lab safety, and relieves operators from repetitive demanding operations [108].

### 4.2. Analytical Determination

#### 4.2.1. Indirect Techniques (Separative Techniques)

Indirect techniques have been widely used and optimized for AFs’ analyses on nuts. Such matrices, like others that undergo AFs’ contamination, are complex and require proper sample preparation before any other manipulation. Once the AFs are extracted and concentrated from the sample, the separation of the analytes can be carried out using different chromatographic methodologies, coupled with a proper detector. It should be noted that the chromatographic and detection techniques are not free from matrix effects; thus, the sample pre-treatment is crucial to limit such an issue. The objective of the chromatographic method is to separate the AFs to allow a proper quantification of the total level of AFs (AFB_1_ + AFB_2_ + AFG_1_ + AFG_2_) and/or their individual levels.

Any appropriate analytical methodology can be used as long as it is robust, replicable, and reliable [34]. The most common methods for identification and quantification of AFs (and mycotoxins in general) are listed in The Official Methods of Analysis (OMA) AOAC International as well as in the Commission Regulation (EC) No.401/2006 [109]. In the following sections, an overview of the main chromatographic techniques for the identification and quantification of AFs developed during the last decade will be presented.

##### Liquid Chromatography Coupled to Classic Detectors

Considering the chemical properties of AFs, it is not surprising that liquid chromatography (LC) has become the most chosen technique for their quantitative analysis. In the 1970s, there was increasing interest in LC-based analysis of AFs, with the expectation that LC would surpass thin layer chromatography (TLC) for routine testing thanks to faster separations with better accuracy and precision. Normal-phase (NP) LC, coupled with UV or DAD, was initially used. This approach was challenging and not free from issues due to the technical limitations of the instruments at that time and the mid-polar nature of AFs, often resulting in overlapping chromatographic peaks. Some improvements have been made over time, but starting from the 1980s, reversed-phase (RP)-LC mode has gradually replaced NP-LC for the analysis of AFs [51], even if NP-LC is still sporadically used in combination with immunoaffinity columns [110].

The physical properties of AFs make them good candidates also for hydrophilic interaction liquid chromatography (HILIC) separations. HILIC is an LC mode mostly used for the analysis of highly hydrophilic, ionic, and polar compounds. Like NP-LC, HILIC uses polar stationary phases (such as silica or amino), while the mobile phase is like those used in RP-LC (mainly a combination of acetonitrile and water) but with a higher organic proportion. Due to this higher organic solvent content compared to RP-LC, HILIC may enhance signal intensities by improving MS efficiency [111]. Nevertheless, only a few applications had been reported in the literature and none of them regarding the analysis of AFs in nuts [112,113].

In RP-LC, AFs are generally analysed using mixtures of water, methanol, and/or acetonitrile as mobile phases. Additionally, AFs are fluorescent, giving a great advantage for detection after separation. Indeed, AFB_1_ and AFB_2_ exhibit strong blue fluorescence (425 nm) when exposed to a 365 nm excitation wavelength, while AFG_1_ and AFG_2_ present green fluorescence (450 nm) [114]. Therefore, RP-LC is often coupled to a fluorescence detector (FLD) for this type of analysis. Nevertheless, coupling RP-LC with this detector presents some challenges. Although HPLC and UHPLC can provide great resolution for the separation of AFs, their fluorescence emission can be considerably quenched by aqueous mobile phases used in RP-LC mode. This limitation could restrain the detection of AFs at sub-ppb levels in the matrix of interest. Commonly, when an FLD is used for AFs detection, either a pre-column or a post-column derivatization step is required to enhance fluorescence. Hence, other common detectors such as ultraviolet (UV) and diode array detector (DAD) are also often employed [115].

##### AFs Derivatization

To overcome the problem regarding quenching when using an FLD, a pre-column derivatization step involving the reaction of AFB_1_ or AFG_1_ with trifluoroacetic acid (TFA) has been widely applied, obtaining, respectively, AFB_2_a and AFG_2_a. This approach is feasible and provides a fluorescing product. The considered product is the result of the hydration of the double bond in the dihydrofuran portion of the considered AF. However, the pre-column derivatization with TFA has several disadvantages, such as long reaction time and low stability of the derivative products, which gradually degrade into non-fluorescing compounds [116]. This approach improved the detectability of AFB_1_ and AFG_2_ by a factor up to 55, whereas AFG_2_ and AFB_2_ are unaffected. AFB_2_ and AFG_2_ have a native higher fluorescence when compared to AFB_1_ and AFG_1_, whose fluorescence is strongly solvent-dependent [117]. Moreover, a pre-column derivatization process introduces an additional step in the analysis, making post-column derivatization more desirable since it can be more easily installed online with the LC system [118]. To limit the issues of pre-column derivatization, TFA as a derivatized agent can also be used in post-column derivatization mode [119].

Post-column derivatization procedures for AFs involve mainly three approaches: (i) iodination, (ii) bromination, and (iii) photochemical derivatization.

i. Iodination was the first post-column derivatization approach implemented back in 1979 when it was noticed that iodination of the double bond of AFB_1_ and AFG_1_ provides products with enhanced fluorescence. Although the optimization of this methodology, regarding different parameters such as time of reaction, reaction temperature, iodine reagent flow rates, and iodine concentration, allowed the development of widely accepted post-column derivatization procedures, it has been rarely applied in the last decade, leaving room for other derivatization procedures [51,120].
ii. The bromination approach can be achieved with either pyridinyl hydrobromide perbromide (PBPB) or with an electrochemical cell (KobraCell) where KBr is added to the mobile phase. During the last decade, this procedure has almost replaced the previous one becoming the first choice as a post-column derivatization approach, particularly considering the AFs determination in nut matrices [107,121,122].
iii. Photochemical derivatization is based on the reaction of AFs with the water contained within the mobile phase, thanks to post-column UV irradiation. This derivatization determines the formation of hemiacetals, compounds similar to those obtained by TFA derivatization, showing enhanced fluorescence. Moreover, this approach is free from the use of other chemicals to obtain the desired derivatives [123]. However, no application has been reported in nuts matrices yet. A critical review of the pros and cons of LC-based techniques hyphenated to post-column derivatization approaches have been recently published by Zacharis et al. [123].

##### Liquid Chromatography Coupled with Mass Spectrometry (LC-MS)

Due to the continuous improvement in analytical instrumentations, the current selectivity and sensitivity of MS technology made this technique the golden standard for the detection of AFs, in particular when coupled with LC-based techniques [124,125]. Indeed, LC-MS can eliminate the derivatization step required to enhance the fluorescence activity [126]. Nevertheless, during an LC-MS analysis, the signal of AFs could be drastically suppressed because of co-eluting matrix components.

Some MS-based techniques have been developed to avoid the clean-up step and directly inject the sample into the instrument after a single liquid extraction, thus reducing sample loss and shortening the analysis time. Despite the attractive simplicity of these procedures, many authors assert that a clean-up step prior to analysis should be mandatory to avoid ionization suppression due to the matrix effect [127]. On the other hand, attention must be paid to avoid the presence of exogenous substances introduced in the sample during the sample preparation step, which may also lead to ionization suppression. Moreover, another method that proved to reduce ion suppression is the dilution of the sample extract to reduce the matrix effect. In addition, other techniques applied to compensate for the matrix effect are external calibration using matrix-matched samples, standard addition, and/or internal standard [128,129,130].

The simplest approach, to overcome the matrix effect, is a dilution of the sample extract in a solvent, but since it diminishes the amount of analyte introduced in the system, this is only a viable option when the required LOQ can still be achieved [130,131]. Sample dilution or a reduction in the injection volume could be inappropriate for trace analysis such as AFs due to the increase in the limit of detection. For instance, this could be an issue for the determination of AFM_1_ in milk, since the FDA and EU set maximum levels of AFs in milk at 0.5 ppb and 0.05 ppb, respectively [11,132]. However, even if it has never been reported yet for AFs analysis, it is important to mention that dilution could lead to an increment of the target signal(s) in trace analysis since the ion suppression effect of the interferences may not be linearly correlated with their concentration in the matrix; thus, the dilution effect should be experimentally tested [133].

External calibration using matrix-matched standards has been used for mycotoxins in general, including AFs, for the analysis of different food commodities [42,134,135,136,137]. Due to the complexity of the matrix of interest, this approach is not always feasible since it relies on the availability of blank samples. Additionally, the blank matrix must be as similar as possible in composition to the sample, or it will fail to compensate for the ion suppression [128,138]. External calibration without using matrix-matched standards (pure solvent) has also been used for the analysis of AFs in different food commodities, including nuts [138].

Internal standard addition is a suitable method for compensating ion suppression [128] since any matrix components co-eluting with the analyte will undergo the same condition analysis of the internal standard, allowing the analyte-to-internal standard response ratio to compensate for any ion suppression that may be present. It is also superior to matrix-matched calibration since the compensation is physically performed in each sample, thus having the same matrix [131,139]. The internal standard can be a compound similar to the analyte, a structure analogue, or an isotopically labelled compound. Regarding this last point, a strategy used to compensate for the matrix effect is the stable isotope dilution assay (SIDA), based on isotopically labelled internal calibration. In this way, analyte response variation is compensated by the isotopologue ratio, making it a great strategy when target analysis is performed, and analytical standards are commercially available [54]. This is the case for AFs, where 13C-AFs and deuterated AFs are readily available. Among these, 13C-labelled AFs are preferred to deuterated ones to guarantee co-elution with their natural analogues.

Among the different atmospheric pressure interfaces (APIs), the electrospray ionization (ESI) source is the most used for the AFs determination by LC-MS since it is very effective to generate protonated molecular ions ([M+H]+). Recently, Mateus et al. developed and validated a semi-targeted methodology for the analysis of AFs and other mycotoxins in pistachio nuts based on QuEChERS followed by RP-UHPLC-ESI(+)Q-time-of-flight(TOF)MS operating in full-scan mode. The LOD achieved for AFs was in a range from 0.125 to 0.25 µg/kg, which is lower than the maximum levels in nuts regulated by the EU and it was obtained without the necessity of MS/MS experiments [79]. Additionally, other API sources such as atmospheric pressure photoionization source (APPI) and atmospheric pressure chemical ionization (APCI) have been successfully applied for the sensitive LC-MS AFs determination [140]. APCI and APPI can generate in-source fragmentation ions similar to those generated by ESI-MS/MS [140].

Applying MS/MS mode, a particular m/z value is selected from the mass spectrum and directed into a collision cell (CC) containing a neutral gas (e.g., argon). The excited gas inside the CC collides with the ion(s) vibrationally, leading to fragmentation. This process is known as collision-induced dissociation (CID). Other MS/MS induced fragmentation approaches include electron capture and electron transfer dissociation, surface-induced-dissociation, and photodissociation. In general, in AFs investigation, MS/MS can be operated in: (i) product ion-scanning (PI) mode (MS1 in SIM or EIC mode and MS2 in scan mode), (ii) precursor ion-scanning mode (MS1 in scan mode and MS2 in SIM or EIC mode), (iii) neutral-loss scanning mode (MS1 and MS2 in scan mode), and (iv) reaction monitoring mode (MS1 and MS2 in SIM and/or EIC mode) [138].

The most popular MS technology in LC-MS for AFs determination is the triple quadrupole (QQQ)-MS with ESI(+/−) interface, operating in multiple reaction monitoring (MRM) mode. This is mostly due to the higher sensitivity of QQQ-MS compared to other MS technologies for trace target analysis. Recently, Demirhan et al. used an LC-ESI(+/−)-QQQ-MS/MS methodology to identify and quantify total AFs (along with other 8 hazardous mycotoxins) in 80 peanut butter and hazelnut butter samples [141]. The recovery values reported were above 94.62% and RSD (%) values were below 4.87 for all AFs. Interestingly, the 52.5% of peanut butter samples analysed exceeded the maximum level for AFB1 set by the Turkish Food Codex (TFC) at 5 ng/kg; moreover, 30% of the aforementioned samples exceeded the maximum level of total AFs, set by the TFC at 10 ng/kg. From their result, it is clear that constant monitoring of risky foods is mandatory to ensure public health. Despite the interesting application of their method, and the results obtained, no easily accessible information regarding the material used (i.e., LC mobile phases, chromatographic column, and sample preparation reagents) is reported.

Other LC-MS/MS technologies have been successfully applied [142]. RP-LC-ESI(+/−)-Q-ion trap(IT)MS/MS methodology operating in MRM mode has been applied for a multi-mycotoxin screening, including AFs, in food and food-related products (including nuts) [42,134,137,143,144,145]. RP-UHPLC-ESI(+/−)-Q-Orbitrap-HRMS/MS in combination with a sample preparation method based on QuEChERS has been applied for multi-mycotoxins determination, including AFs in edible nuts [80].

Despite the great potential and reliability of MS/MS for quantitative analysis, only a few routine laboratories are prone to invest in LC-MS/MS as the primary methodology for routine testing. The costs associated with an MS/MS instrument are not negligible and analytical expertise is required [127].

#### 4.2.2. Other Separation Techniques

##### Multidimensional Liquid Chromatography (2D-LC)

As already mentioned, one of the most challenging problems in LC-MS analysis of AFs is the matrix effect. Aside from the already discussed strategies to control and compensate for this issue such as dilution, external calibration, or internal standard addition, 2D-LC was suggested as a possible highly effective separation method to compensate for LC-MS and LC-MS/MS signal suppression effects for quantitative characterization of complex matrix samples [146].

The use of 2D-LC in both heart-cut and comprehensive (LC × LC) modes benefits from the complementary selectivity of two different stationary phases (located in 1D and 2D, respectively). Briefly, in heart-cut 2D-LC, part of the eluate is transferred from the first (1D) to the second dimension (2D), while in LC × LC, the whole eluate is transferred from 1D to 2D by means of a 2-position switching valve modulator. The details on the operation modes of multidimensional LC are out of the scope of this review and interested readers are invited to consult the dedicated references [147,148,149].

Applications of 2D-LC for AFs analysis have been reported in not many publications and all of them used heart-cut mode as more frequently applied in the field of target analysis. In contrast, comprehensive chromatography is more common in metabolomics, proteomics, and other non-target determinations of very complex, matrix-rich samples [150,151]. It is worth mentioning that the use of 2D-LC has contributed to reduce the matrix effect in different matrices such as cereals and tobacco [152,153]. However, none of the published works have considered nuts as a matrix of interest so far.

##### Gas Chromatography (GC)

AFs are non-volatile semi-polar compounds, with a melting point ranging from 238.5 °C (AFG_2_) to 287 °C (AFG_1_) [154]. Thus, the analysis of these compounds by gas chromatography (GC) did not attract the attention of the scientific community. Although the scope of this review is to present the latest developments in the analysis of AFs, the authors consider that it is worth having a brief overview of the GC analysis performed in this field.

GC analysis of AFs became feasible with the arrival of fused silica capillary columns. In 1984, Rosen et al. were the first to use a GC-MS approach as a confirmatory method for AFB_1_ and AFB_2_ in peanuts [155]. Unfortunately, the method was not developed for the quantitation of total AFs. In the same year, Trucksess et al. conducted a recovery experiment for total AFs on corn and peanuts also using GC-MS [156]. Some years later, a further qualitative study reported by Goto et al. showed the possibility to detect total AFs using an on-column injection method in GC-FID [157]. Currently, due to the rise of LC-FLD and LC-MS for AFs quantitation in foodstuff, these GC approaches have been set aside. Nevertheless, interesting and promising indirect approaches investigating the VOCs profile in contaminated/non-contaminated nuts by using GC-based techniques and chemometrics have been presented, as discussed in the sample preparation section [95,96].

##### Supercritical Fluid Chromatography (SFC)

**Figure 5:**
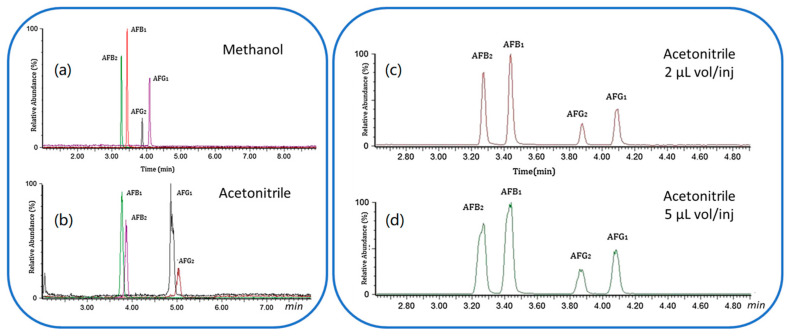
On the left, the effect of the modifier on the AFs elution order, (**a**) methanol and (**b**) acetonitrile; on the right, the effect of different volume injection on the peak shape using only acetonitrile, (**c**) 2 µL (**d**) 4 µL. Modified and reprinted with the permission of [158].

The mid-polar nature of AFs does not make them suitable analytes for SFC. However, the addition of a polar organic co-solvent such as methanol to CO_2_ compensates for the mismatch in polarity between AFs and CO2, allowing to perform a proper separation of AFs. To the best of our knowledge, the only publication during the last decade regarding the use of SFC for the analysis of AFs (in an edible oil) has been reported by Lei et al. Edible oil was spiked with isotope labelled AF standards, and the extract was directly loaded to an SFC system and separated with a CO_2_:methanol gradient elution, from 2% to 20% methanol in 5 min. The SFC was coupled with an ESI(+)-QQQ-MS/MS system. A post-column make-up flow was introduced after SFC separation to facilitate the MS performance, and the mixture was analysed in MS/MS mode. Both methanol and acetonitrile have been used as modifiers and different volume injections have been tested to reduce peak broadening. Figure 5 shows how the selection of the modifier can affect the AFs selectivity (on the left) and the effect of sample injection volume using the same solvent can affect the peak shape (on the right). This method resulted in good recovery, ranging from 93 to 104%. The LOQs for the AFs were 0.05–0.12 μg/L, while the RSDs were lower than 8.5% [158]. In the same year, an online SFE-SFC-ESI(+)QQQ-MS/MS methodology was proposed to extract and analyse AFB_1_ and AFB_2_ in peanut butter [68].

#### 4.2.3. Direct Techniques

Although many of the methods for analysis of AFs in food that have been discussed in the previous sections can be reliable and precise, they require well-equipped laboratory and skilled personnel. Moreover, they are labour intensive, destructive, and in many cases considered expensive [159]. An interesting alternative to these methods is direct analysis techniques, where the sample pre-treatment is reduced to a minimum. These techniques can provide a quicker response with acceptable results even for quantitation, respecting the official guidance for the analysis of AFs. Additionally, some of these direct techniques can be used directly in the field, without bringing samples to the laboratory, and they can be non-destructive. In the next sections, we discuss the most popular of these techniques in this field. However, some others present great potential, although not largely in use in this area. Further information on these other techniques can be found elsewhere [127,160,161].

##### Direct MS-Based Techniques

Matrix-assisted laser desorption/ionisation (MALDI) and direct analysis in real time (DART) coupled to MS can be used for the analysis of food matrices without the necessity of chromatographic separation. In MALDI-MS, the analyte is co-crystallised with an ultraviolet-adsorbing organic acid matrix. Then, this organic acid matrix is vaporised by laser radiation, carrying the analyte with it. To the best of our knowledge, only few scientific contributions applying this technique in nut samples have been reported [162,163]. The main application of MALDI technology in this field has been in proteomic studies to discriminate between aflatoxigenic and non-aflatoxigenic fungi in nut samples [164,165,166].

In the case of DART, condensed-phase analytes are thermally desorbed by means of a hot plasma that ionises the analytes. Ions resulting from the analyte ionization processes are sent into the atmospheric pressure interface of the MS instruments for subsequent mass detection [167,168]. As for MALDI, complex matrices can be challenging due to the lack of chromatographic separation. The use of DART with high-mass resolution and tandem MS can lead to greater selectivity and specificity. Such accuracy is often necessary to compensate for minimal sample preparation and a lack of chromatographic separation [169]. DART ionisation coupled with a high-resolution MS (HRMS) has been successfully applied to investigate AFs in corn, milk, and other food/feed matrices [169,170]. However, its use for the investigation of AFs in nut matrices has not been reported yet.

##### Enzyme-Linked Immunosorbent Assay–ELISA

Immunoassays have become very popular for everyday screening analysis of AFs thanks to their simplicity and straightforward application [171]. The principle behind enzyme-linked immunosorbent assay (ELISA) relies on antibodies to detect a target antigen using antibody–antigen interactions. Antibodies are fastened on a proper plate or column. When this support is exposed to AFs, the antibodies recognize epitopes of AFs to form a complex. The complex is then exposed to a chromogenic substance that creates a signal from the interaction in the form of light, electricity, or another measurable parameter. The results are almost immediate; therefore, food commodities subjected to AFs analysis can be easily processed without delay. ELISA tests can provide qualitative or quantitative results. Qualitative ELISA provides a simple positive/negative response for a sample. In quantitative ELISA, the optical density or fluorescent units of the sample is interpolated into a standard curve, which is typically a serial dilution of the target [172].

Mycotoxins rapid tests are combined with a simple sample preparation procedure such as extraction with methanol:water (or buffer), filtering, and dilution with buffer. This procedure is mandatory since the matrix effect is a significant problem affecting the results [173]. Nevertheless, ELISA provides comparable LODs with those of instrumental methods. In some publications comparing ELISA with HPLC-FLD or HPLC-MS/MS, a strong correlation between these methods is reported. For instance, when comparing ELISA and LC-MS/MS for the quantitation of AFB_1_ in corn, Stefanovic et al. reported a high level of agreement in the determination of AFB_1_ (R^2^ = 0.994) [174]. In a similar study investigating the level of AFM_1_ in milk, Kos et al. obtained correlation coefficients higher than 0.9, indicating a strong correlation between HPLC-MS/MS and ELISA [175].

Nowadays, different ELISA kits are commercially available and are used for the detection and quantification (using an ELISA plate reader) of total AFs in nuts, as much as other food commodities [176,177,178,179]. It is worth mentioning that AOAC International approved two ELISA-based screening procedures for qualitative determination of AFB_1_ in roasted peanuts and AFB_1_, AFB_2_, and AFG_2_ in peanut butter [180,181].

##### Lateral Flow Device

Lateral flow device, also known as lateral flow test, is a fast and easy-to-handle immunoassay test that can be both qualitative, with a defined cut-off level, or quantitative when used with a photometric strip reader. The lateral flow device operates on the same principle as ELISA [182]. Briefly, a liquid sample (extract) is loaded on a strip made of nitrocellulose membrane and the capillary forces drive the sample along the strip. During the migration, the antigens interact with antibodies (usually colloidal gold-conjugated anti-mycotoxin antibodies) present along the strips. This whole complex migrates along the membrane and binds with the secondary antibodies on the test line. The further migration leads antigen-free antibodies to interact with anti-mouse antibodies on the control line. In the presence of target antigens, both control and test lines will turn red, otherwise only the control line will appear as red. Colloidal gold conjugated antibodies are the most used since the 40 nm colloidal gold particles have a deep red colour thanks to plasmon resonance effects, which is exploited for test strip signaling [183]. In order to convert this visual response to an analytical concentration, diagnostic devices, known as lateral flow readers, are usually applied. These readers use image processing and specifically designed algorithms to give a proper response [184]. Li et al. developed a fluorometric lateral flow immunoassay (LFIA) for rapid screening analysis of AFB_1_ and other mycotoxins in 17 naturally contaminated feedstuff (including nuts) and cereals samples. To verify the accuracy of the screening results for the cereals and feedstuff samples, the results were confirmed by LC–MS/MS showing a correlation value for AFB_1_ equal to 0.97 [185]. Chen et al. developed a quantitative multiplex LFIA for the simultaneous determination of mycotoxins (including AFB_1_) in corn, rice, and peanuts. The LOD for AFB_1_ obtained was 0.10–0.13 μg/kg, far below the regulatory limits set by the European Commission. At the spiked concentration of 0.5–10.0 μg/kg, the mean recovery of AFB_1_ ranged from 86.2 to 114.5% with coefficients of variation less than 16.7%, showing that the LFIA could be used for routine monitoring of AFB_1_ contamination [186].

##### Immunoaffinity Column, Direct Fluorescence Measurement

As already discussed in the sample preparation section, IACs became widely used and commercially available for the analysis of AFs. It is worth mentioning that this approach is used not only to have a clean extract ready to load on an LC system, but also to have a direct fluorescence measurement of the extract, and hence a quantitative determination of AFs. When it comes to nuts, this direct analysis is registered as AOAC Official Method 991.31 [57]. This method uses an Aflatest^®^ IAC column from VICAM. For quantitation, the methanol eluate from the IAC is collected in a fluorometer cuvette and immediately subjected to fluorometric determination. The fluorescence detector should have a 360 nm excitation length and >420 nm cut-off emission filter and it must undergo calibration with proper standards.

Although the IAC-FLD methodology is more than 20 years old, this approach remains a useful method for screening everyday samples thanks to the short analysis time, the relatively low cost of a fluorometer, and the lower variable costs of the method. Nevertheless, a recent study by Hazef et al. for the detection of AFB_1_, compared an in-house developed ELISA technique versus Aflatest^®^ IAC and HPLC-FLD [187]. Their results showed that the IAC-FLD was less sensitive in a range between two and three orders of magnitude than HPLC-FLD analysis for AFB_1_ in several samples of peanuts, flour, and milk powder.

##### Image Processing

Image processing is defined as the use of computer algorithms applied to the analysis of images. This approach aims to get an enhanced image or extract useful information from it [188]. Image processing methods have been already investigated to detect contamination by AF-producing fungi because they potentially rapidly detect and physically identify, for removal, contaminated products [189]. Different techniques based on image processing have been developed and applied for AFs detection in nuts. These techniques include near-infrared spectroscopy (NIRS), mid-infrared spectroscopy (MIRS), conventional imaging techniques such as colour imaging (CI) and hyperspectral imaging (HIS), and fluorescence spectroscopy/fluorescence imaging (FS/FI). The results obtained can be processed with different chemometrics algorithms [190,191,192]. A detailed overview of the operating mechanisms of these techniques is out of the scope of the present review, and the topic has been already reviewed [192].

Among these techniques, FS/FI and HIS are the most studied and applied in this field since they could be implemented for on-line analysis of food matrices [193,194,195,196]. FS and FI take advantage of the natural fluorescence of AFs when these are exposed to UV light at 365 nm. Specifically, class B AFs emit fluorescence in the bright-blue region of the spectrum (425–480 nm), while class G AFs emit fluorescence in the blue-green range (480–500 nm) [197]. In FS, the specific wavelength emitted by AFs can be detected by either single-line excitation and dispersion of the entire fluorescence emission spectrum or by tuning the excitation source over a wide wavelength range and detecting the entire spectrum of emitted light with a broadband detector [192]. As a non-destructive and cost-efficient technique, FS has been extensively exploited for mycotoxin determination in various agricultural commodities [198]. Instead, FI is based on the acquisition of images, through a camera, when the matrix of interest is exposed to UV light. The image thus obtained is elaborated in post-acquisition to detect anomalies in the sample [194]. Different from the traditional spectroscopic techniques offering only spectral information, the HSI technique has emerged to provide not only spectral information, but also spatial images as provided by the CI technique, which enables HSI to characterize fungi and AFs reliably [192]. These techniques are still a novelty and need further development to overcome some limitations, such as high LOD, background interferences, and environmental factors [199]. With the rise of artificial intelligence algorithms during the last few years, some interesting applications involving image processing and artificial neural networks have been implemented for AFs detection in nuts. For instance, in 2021, Gao et al. used rapid detection based on hyperspectral imaging with 1D-convolution neural networks at the pixel level. After the acquisition of hyperspectral images, of single kernels, the data were processed with the developed neural network to classify whether a pixel contains AFs. The authors claim the difficulty in estimating the content of AF in one pixel. Therefore, if the AFB_1_ concentration was >0 ppb in one pixel, this pixel was marked as contaminated. As a consequence, this paper only detects whether AF is contained in one pixel of the peanut surface. Their results showed the highest accuracy of 96.35% for nuts over other samples tested (corn) [200,201]. Soemantri et al. reported even higher accuracy for corn (an average accuracy of 99%) [202].

**Figure 6:**
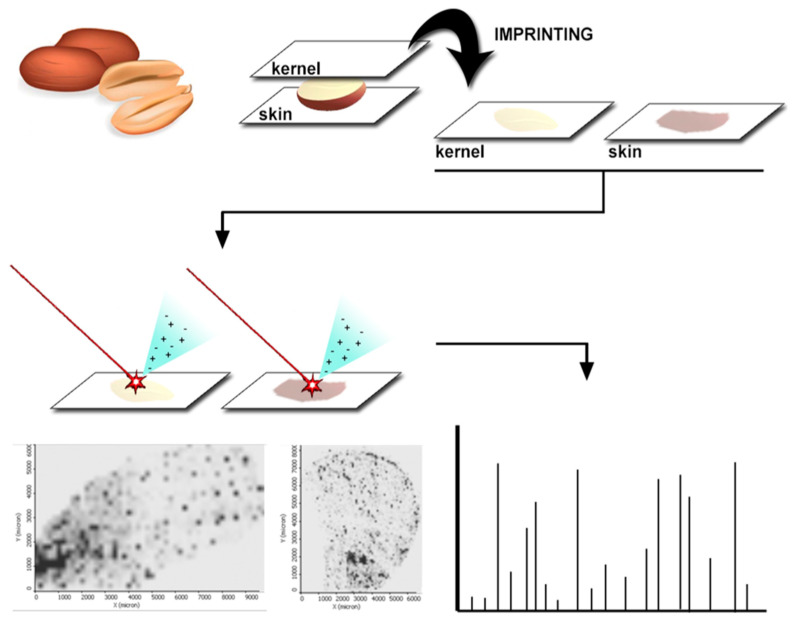
Workflow of the SPILDI-MSI experiments for compound identification in peanut skin and kernel. Cross-sections of the kernel and the skin are imprinted in a TLC plate and then sent for MSI analysis. Reprinted with the permission of [163].

A different image processing technology using MALDI-HRMS has been successfully applied by Oliveira et al. The authors used a silica plate imprinting laser desorption/ionisation mass spectrometry imaging (SPILDI-MSI) for the detection of AFs in nuts. This approach is solvent-free and does not require any extraction or complex separation steps. The SPILDI experiment consisted in pressing the nut’s skin, removed from the kernel, and a thin transversal section of the kernel (1 mm) between two silica 60 TLC plates for 5 min (see Figure 6). These samples were then analysed by MALDI(+)-linear(L)IT-Orbitrap-HRMS/MS imaging. The authors claimed it was possible to observe that all types of AFs were present, even deeper into the internal region of the kernels. The data acquired were supported by the comparison with the MS/MS fragmentation pattern of standards. This application showed an excellent qualitative assessment of AFs both in the peanut skin and kernel, bringing precise tracking of the fungal contamination [163].

## 5. Conclusions and Future Perspectives

It is clear that the sample pre-treatment is a key step in the determination of AFs. However, there is no single approach that can fit all cases. Although some official recommendations exist, the choice of one pre-treatment and sampling over the other is linked to the specific application, depending on sample type, available sample amounts, and the technical resources that can be used. Interestingly, greener alternatives to classical pre-treatments such as MAE proved useful, but their potential remains largely underused for routine testing. In terms of analytical techniques, the use of HPLC prevails due to its simplicity and availability, and the straightforward application to the extracts obtained with the most common pre-treatment approaches. Different detection techniques have evolved from FLD with derivatization to MS, which has become the working standard in this field. Nevertheless, sometimes the use of MS for routine analysis is rather limited due to its operating cost. Moreover, although LC-MS can achieve the required levels of sensitivity, the matrix effect may be an important drawback. This leads to the exploration of alternatives to overcome this issue, such as multidimensional chromatography. However, despite the great potential of the increased separation power of this approach, this remains a novelty and its application to routine analysis is uncertain.

Alternatively, to avoid the cumbersome and labour-intensive sample preparation, the use of direct analytical approaches has gained interest. In this area, the use of ELISA or lateral flow devices had gained popularity thanks to their simplicity for screening multiple samples with minimal preparations, along with techniques that can be applied directly to the samples, such as direct MS or hyperspectral imaging. The latter is of particular interest and fits potential applications with handheld devices that would not require off-site laboratory work. Nevertheless, their application is not yet widespread.

Certainly, the new toxicological information that is obtained by the newer methodologies will continue to improve the understanding of the risk of these compounds, and limitations in their content may become stricter over time. Therefore, there will be an increasing need for more reliable and sensitive methods. Moreover, given the large size of the studied samples and the clear impact of the sampling and pre-treatment on the variability of the obtained results, sampling strategies may need to be reviewed in light of the new findings, and better pre-treatments may be required. Finally, it should be noted that ease of use should be considered a key element in the development of new technologies for quality control in this area to enable proper control of these contaminants worldwide.

## Data Availability

Not applicable.
